# AbDiver: a tool to explore the natural antibody landscape to aid therapeutic design

**DOI:** 10.1093/bioinformatics/btac151

**Published:** 2022-03-11

**Authors:** Jakub Młokosiewicz, Piotr Deszyński, Wiktoria Wilman, Igor Jaszczyszyn, Rajkumar Ganesan, Aleksandr Kovaltsuk, Jinwoo Leem, Jacob D Galson, Konrad Krawczyk

**Affiliations:** NaturalAntibody, 71-064 Szczecin, Poland; NaturalAntibody, 71-064 Szczecin, Poland; NaturalAntibody, 71-064 Szczecin, Poland; NaturalAntibody, 71-064 Szczecin, Poland; Medical University of Warsaw, 02-091 Warsaw, Poland; Alector, South San Francisco, CA 94080, USA; LabGenius, SE16 4DG London, UK; Alchemab Therapeutics Ltd, N1C 4AX London, UK; Alchemab Therapeutics Ltd, N1C 4AX London, UK; NaturalAntibody, 71-064 Szczecin, Poland

## Abstract

**Motivation:**

Rational design of therapeutic antibodies can be improved by harnessing the natural sequence diversity of these molecules. Our understanding of the diversity of antibodies has recently been greatly facilitated through the deposition of hundreds of millions of human antibody sequences in next-generation sequencing (NGS) repositories. Contrasting a query therapeutic antibody sequence to naturally observed diversity in similar antibody sequences from NGS can provide a mutational roadmap for antibody engineers designing biotherapeutics. Because of the sheer scale of the antibody NGS datasets, performing queries across them is computationally challenging.

**Results:**

To facilitate harnessing antibody NGS data, we developed AbDiver (http://naturalantibody.com/abdiver), a free portal allowing users to compare their query sequences to those observed in the natural repertoires. AbDiver offers three antibody-specific use-cases: (i) compare a query antibody to positional variability statistics precomputed from multiple independent studies, (ii) retrieve close full variable sequence matches to a query antibody and (iii) retrieve CDR3 or clonotype matches to a query antibody. We applied our system to a set of 742 therapeutic antibodies, demonstrating that for each use-case our system can retrieve relevant results for most sequences. AbDiver facilitates the navigation of vast antibody mutation space for the purpose of rational therapeutic antibody design.

**Availability and implementation:**

AbDiver is freely accessible at http://naturalantibody.com/abdiver.

**Supplementary information:**

Supplementary data are available at *Bioinformatics* online.

## 1 Introduction

Monoclonal antibodies are the largest class of biotherapeutics. Development of successful antibody therapeutics requires selection and engineering of candidate sequences with favorable functional and developability properties. Knowledge of biologically possible mutations at specific positions can be employed to engineer biophysical properties of these molecules (Venkataramani *et al.*, 2020). Next-generation sequencing (NGS) now allows us to capture millions of naturally sourced B cell receptor (BCR) sequences in a single experiment, providing insight into natural antibody diversity (Kovaltsuk *et al.*, 2018).

The richness of NGS data has implications for rational selection and design; models trained on these data have already shown promise for humanization (Marks *et al.*, 2021) and binding prediction (Mason *et al.*, 2021). It has also been shown that close sequence matches to clinically approved antibodies can be found in NGS datasets (Krawczyk *et al.*, 2019), and clinically approved antibodies contain engineered mutations that can be recapitulated using the natural diversity from these datasets (Petersen *et al.*, 2021). Exploration of natural antibody diversity from NGS datasets relative to a candidate therapeutic would therefore facilitate rapid and effective antibody engineering.

The volume of the publicly available antibody NGS data makes investigation of their diversity mostly constrained to time-consuming bioinformatic endeavors. There exist online tools to retrieve antibody sequences from large databases, such as ClonoMatch (Jones *et al.*, 2021), PIRD (Zhang *et al.*, 2019) or the AIRR Data Commons (Christley *et al.*, 2020). Here, we offer an orthogonal service with a therapeutic focus, called AbDiver, that allows users to discover and characterize the natural sequence diversity surrounding a query antibody of interest. We provide three different approaches for performing this mapping (i) annotation of the natural positional diversity statistics on a position-by-position basis for a given query sequence, (ii) finding close matches to a full variable-region sequence and (iii) identifying sequences that would be classed as belonging to the same clonotype as the query sequence (based on CDR3 and germline V/J-gene segments). AbDiver thus provides an accessible way to find a natural reference for a query therapeutic sequence, offering insights for sequence selection and rational design.

## 2 Implementation

*Data*: As the underlying data, we used publicly curated unpaired BCR NGS datasets from the Observed Antibody Space (OAS) (Kovaltsuk *et al.*, 2018). In May 2021, the dataset encompassed 81 studies with 906 933 358 (105 730 531 light chains and 801 202 827 heavy chains) unique BCR sequences numbered according to the IMGT scheme (see Supplementary Material). We envisage updates to the services as more datasets become available. For benchmarking, we used a set of 742 therapeutic antibodies, extending a set from our previous study (Krawczyk *et al.*, 2021). Certain therapeutics were multispecific or contained chain duplicates resulting in 738 unique heavy chains, 707 unique light chains, 686 unique CDRH3s and 573 unique CDRL3s.

**Fig. 1.**
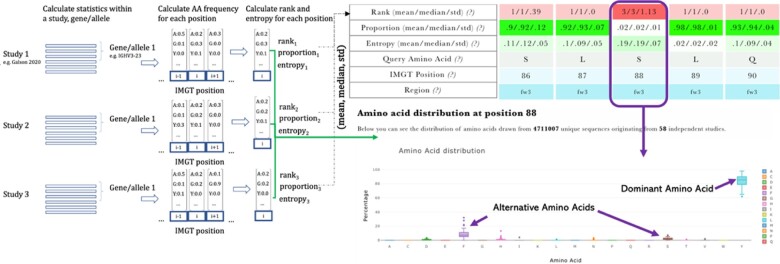
Visualization of the profiling service. Query sequence is compared to the frequency distribution statistics of amino acids of NGS sequences within the same gene or allele. Profiles are calculated for a specific gene or allele. Within each allele or gene, frequencies of amino acids are calculated from IMGT-aligned sequences. The individual study frequencies are used to calculate the mean, median and standard deviation (std) of the amino acid ranks, proportions and entropies which are displayed for each residue. Upon clicking on individual residues, box plot displays positional frequencies aggregated from raw data from individual studies to reflect the observed variability among independent samples

*V-region profiling service*: The AbDiver V-region natural profiling service annotates a query variable-region antibody sequence with the naturally observed amino acid frequency statistics for each position (Fig. 1, Supplementary Material). The frequency statistics are calculated from all antibodies comprising the same combination of V-gene and J-gene. Separate statistics were calculated for genes and their constituent alleles to allow for fine-grained allelic analysis, but also to reflect the ongoing effort in allele annotation (Smakaj *et al.*, 2020). Each IMGT position in each profile contains statistics from amino acid frequencies calculated for each study separately. Amino acid positional frequency for a study was incorporated if it included at least 100 observations at a given position. For each position, we calculated the study-specific Shannon entropy and ranks of the amino acids by frequency. Query sequence sharing the germline genes or alleles of a given profile is then annotated at each IMGT position with the ranks and entropies of the given amino acid, averaged from the ranks and entropies of individual studies. This approach is designed to mitigate the effects of different numbers of sequences, techniques and disease states contributed by different studies, emphasizing frequency commonalities independent of study-specific biases. For benchmarking of the profiling service on 742 therapeutics, please see the Supplementary Material.

*Sequence retrieval service*: We created *k*-mer (*k* = 5) based indexes for CDRs in full variable-region sequences and CDR3s separately. Variable sequence matches are identified based on the same length CDR1, CDR2 with one residue discrepancy allowed for CDR3. Clonotypes are identified on the basis of the same V-gene and CDR3 sequence identity. The matches are presented using IMGT-based Multiple Sequence Alignment (Martin, 2014). Search results are presented using interactive tables highlighting the leading themes in the studies (e.g. studied disease, vaccine) facilitating further exploration of results. For benchmarking of the search service on 742 therapeutics, please see the Supplementary Material.

## 3 Discussion

We created an online portal that facilitates the navigation of natural antibody diversity. We envisage particular application of the service to enable drawing parallels between natural and therapeutic antibodies (Krawczyk *et al.*, 2019) for the purpose of engineering to remove Post-Translational Modification risks while maintaining favorable biophysical properties. For instance, removing a deamidation motif would require one to introduce one of few standard mutations (e.g. NA, QG) and reassess the function of the antibody. AbDiver can identify sequence-similar candidates from natural origin, increasing the chances that function and immunogenicity will not be compromised (Gutiérrez-González *et al.*, 2021). Beyond facilitating liability removal, AbDiver could excavate sequences with potentially better product profiles than the lead therapeutic. Using the CDR3 or clonality search can accelerate the discovery of clones that share therapeutic properties of the query, yet provide alternatives with potentially better product profiles. We hope that AbDiver will enable research-supporting applications to facilitate decision-making in rational design of therapeutics during lead optimization. 

*Financial Support*: none declared.

*Conflict of Interest*: none declared.

## Supplementary Material

btac151_Supplementary_Data
