# Supplementary material for: AbDiver: a tool to explore the natural antibody landscape to aid therapeutic design
Source: Bioinformatics. 2022 Mar 11;38(9):2628–30. doi: 10.1093/bioinformatics/btac151 (PMC9048670; doi:10.1093/bioinformatics/btac151)
Supplement: btac151_Supplementary_Data [file btac151_supplementary_data.pdf]

Supplementary materials for AbDiver – A tool to explore the natural antibody land-scape to aid therapeutic design.

Section 1. Supplementary Methods.

1.1. Data

We employed the Observed Antibody Space database as the source of IMGT-numbered sequences (Kovaltsuk *et al.*, 2018). The data contained herein has the benefit of having been processed using a single assembly & antibody-specific annotation pipeline. The IMGT (Lefranc, 2011) numbering and gene call annotations in OAS are handled by ANARCI (Dunbar and Deane, 2015). Though it is known that employing different gene references can affect the results (Smakaj *et al.*, 2020), we decided to use the OAS-included annotations as the baseline as at the very least it provides the same annotation for all sequences.

Gene calls could pose specific issues within the remit of the therapeutic applications of our platform. We recognize that one might seek a CDR3/clonotype that originated from a custom scaffold, humanized mouse etc. that does not have a clear annotation in the dropdown on our website. In such cases, we suggest that the user perform a calculation themselves which V-gene(s) their scaffold is closest to in terms of sequences identity. Alternatively one can submit the full length sequence to our profiling service which should identify the V-region which can be subsequently used in the CDR3/clonotype search.

Within our search services we allow the user to either choose to find the closest match to either human or mouse or provide a list of organisms for specific gene search. The choice of the organisms is reflecting the bias towards human or mouse sequencing datasets and smaller availability of these coming from other organisms. We expect that as more species reference genes & BCR studies become available, the service will be extended with others. This should include not only canonical antibodies from multiple species, but also specific formats such as single domain antibody as found in camelids (Deszy´nski *et al.*, 2022).

1.2 Variable Profile Calculation

We calculated the immunoglobulin sequence profiles that capture the diversity of these molecules either within genetic or allele context. For each study we identified sequences that belong to either a single gene or allele according to IMGT and after redundancy removal (95% (Steinegger and Söding, 2018)), these served as a basis for profile creation. Within such gene or allele sets from a single study, we aligned the sequences using the IMGT scheme and calculated positional frequencies. We only calculated frequencies for positions that had more than 1,000 entries in an IMGT position. Therefore, each position from a single gene or allele, from a single study, was associated with a 20-entry vector $v_{s,g,i} = (a_1,a_2,a_3\ldots,a_{20})$ where a_i corresponded to frequency of a particular amino acid, s is a particular study, g is particular germline annotation and i is the specific IMGT position. Each vector is then associated with entropy – Shannon entropy calculate from frequencies of the 20 amino acids as well as ordered list of ranks of frequencies with 1 corresponding to the most frequent rank, with ties being assigned same ranks.

In order to avoid biases introduced by different biological conditions, sequencing depths etc. across studies, we chose to calculate ranks and entropies of global positional frequencies across studies. Rank and entropy for a specific gene or allele IMGT position are given as means from all the studies for which we could calculate such vectors. We note that this metric might be afflicted by outliers so in the presentation of frequency distributions, box plots are used that allow the user to examine kernel density first hand. An overview of this entire process is given in Figure 1 in the main manuscript.

Section 2. V-region profile benchmark.

We tested whether the profiling service could find suitable profiles for our 742 therapeutics. A successful profile was arbitrarily defined as requiring at least 10,000 sequences contributing to its calculation (Supplementary Figures 1, 2). Using allele-based profiles there were 688/738 (93.22%) heavy chains and 486/707 (68.74%) light chains where profiles had more than 10,000 OAS sequences. Using the less-stringent gene-based profiles there were 699/738 (94.71%) heavy chains and 496/707 (70.15%) light chains where profiles had more than 10,000 sequences. The smaller number of light chains we can find suitable profiles for results from a skew towards heavy chains in NGS depositions.

We also plotted the number of IMGT framework positions that do not match with top amino acids in identified gene-based (Supplementary Figure 3) and allele-based (Supplementary Figure 4). The higher number of framework positions disagreeing with NGS distribution in the gene-based profiles reflects multiple alleles contributing to these. Our profiles indicated that the majority of therapeutic antibodies contain framework mutations that are not commonly found in naturally occurring antibodies. In total, 191/738 (25.88%) heavy chains and 125/707 (17.68%) light chains from the therapeutic antibodies contained more than five framework mutations not commonly found in naturally occurring antibodies. Therefore, our service identifies profiles for the majority of therapeutic antibodies highlighting non-trivial positional frequency information.

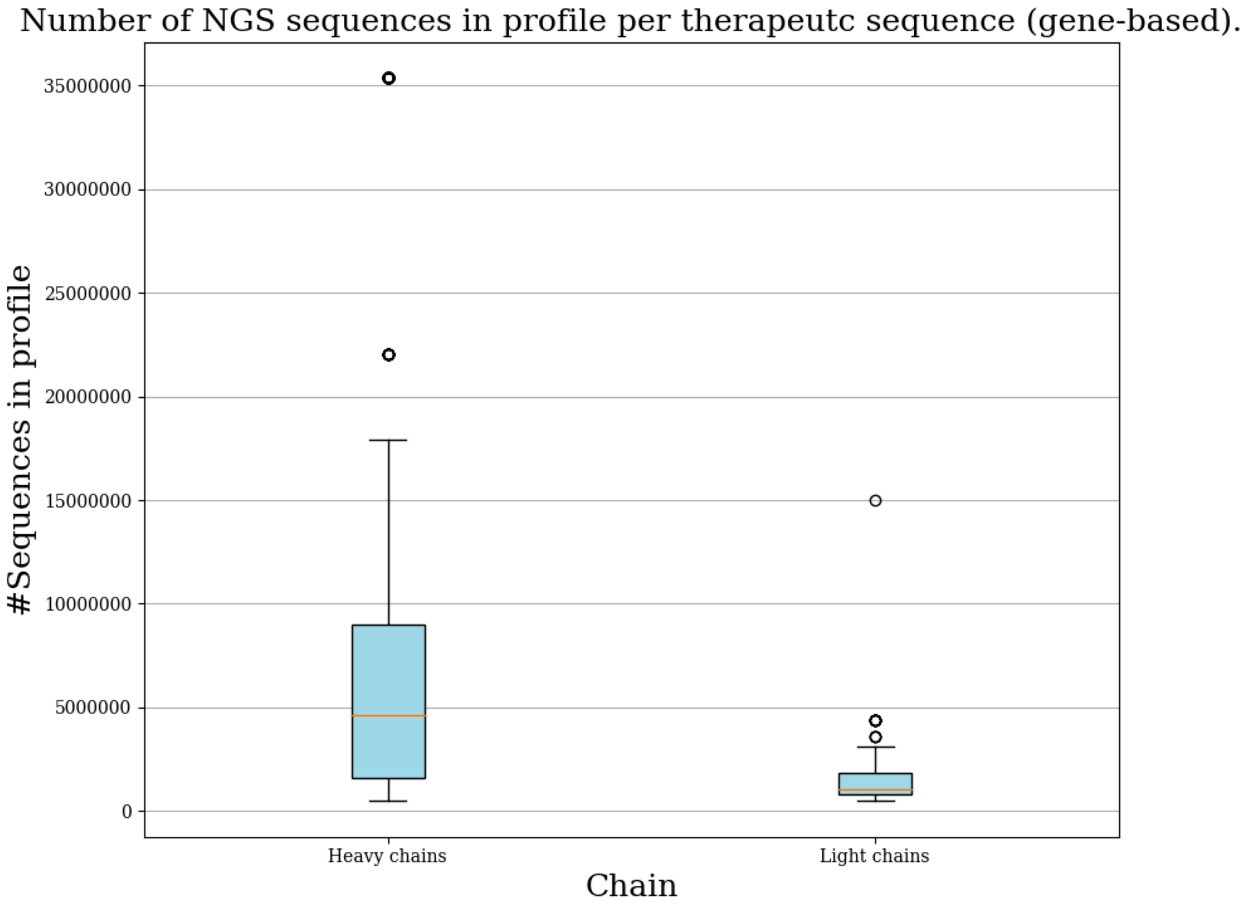

Supplementary Figure 1. Number of sequences in gene profiles per therapeutic sequence. For heavy and light chains in 742 therapeutic antibodies we plotted the number of sequences contributing for a gene-based profile.

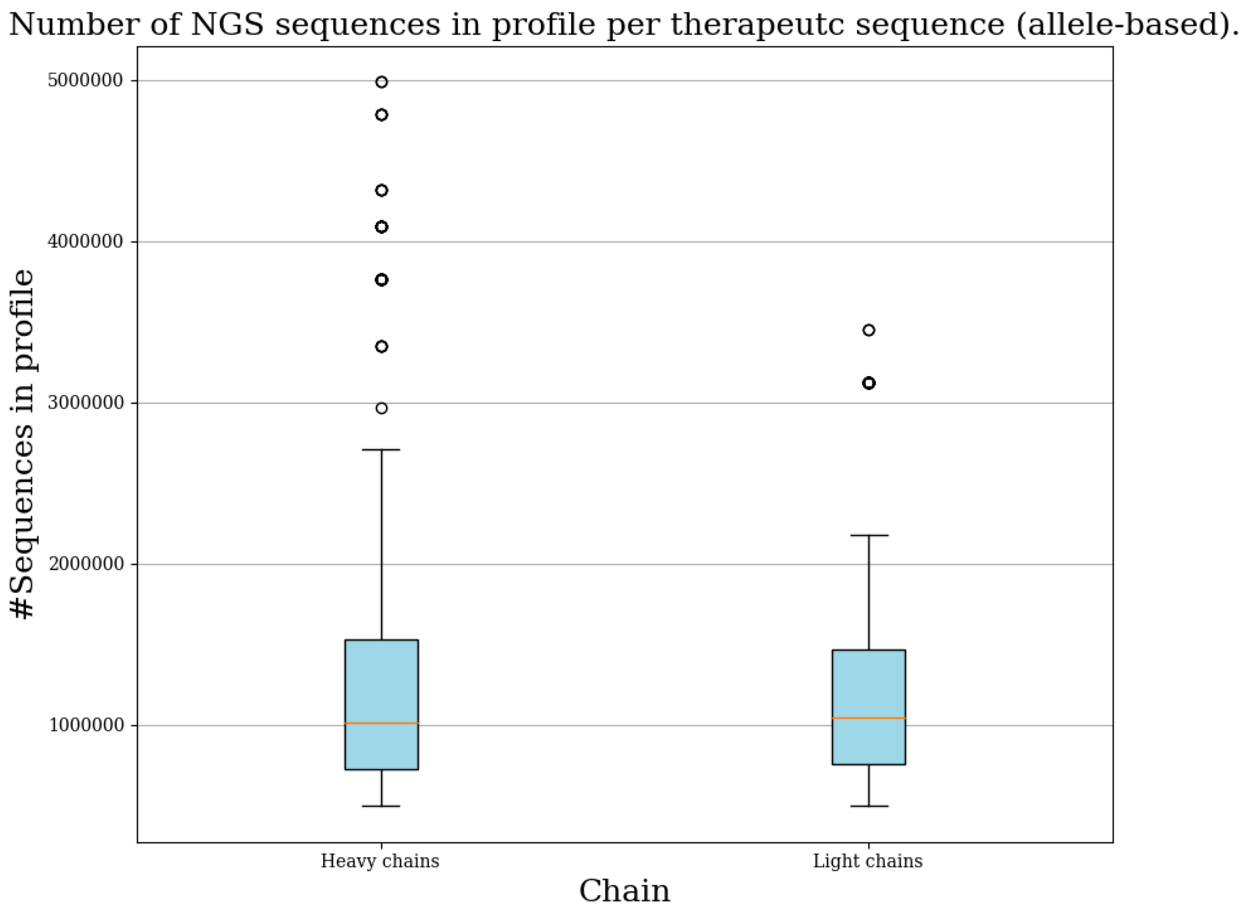

Supplementary Figure 2. Number of sequences in allele profiles per therapeutic sequence. For heavy and light chains in 742 therapeutic antibodies we plotted the number of sequences contributing for a gene-based profile.

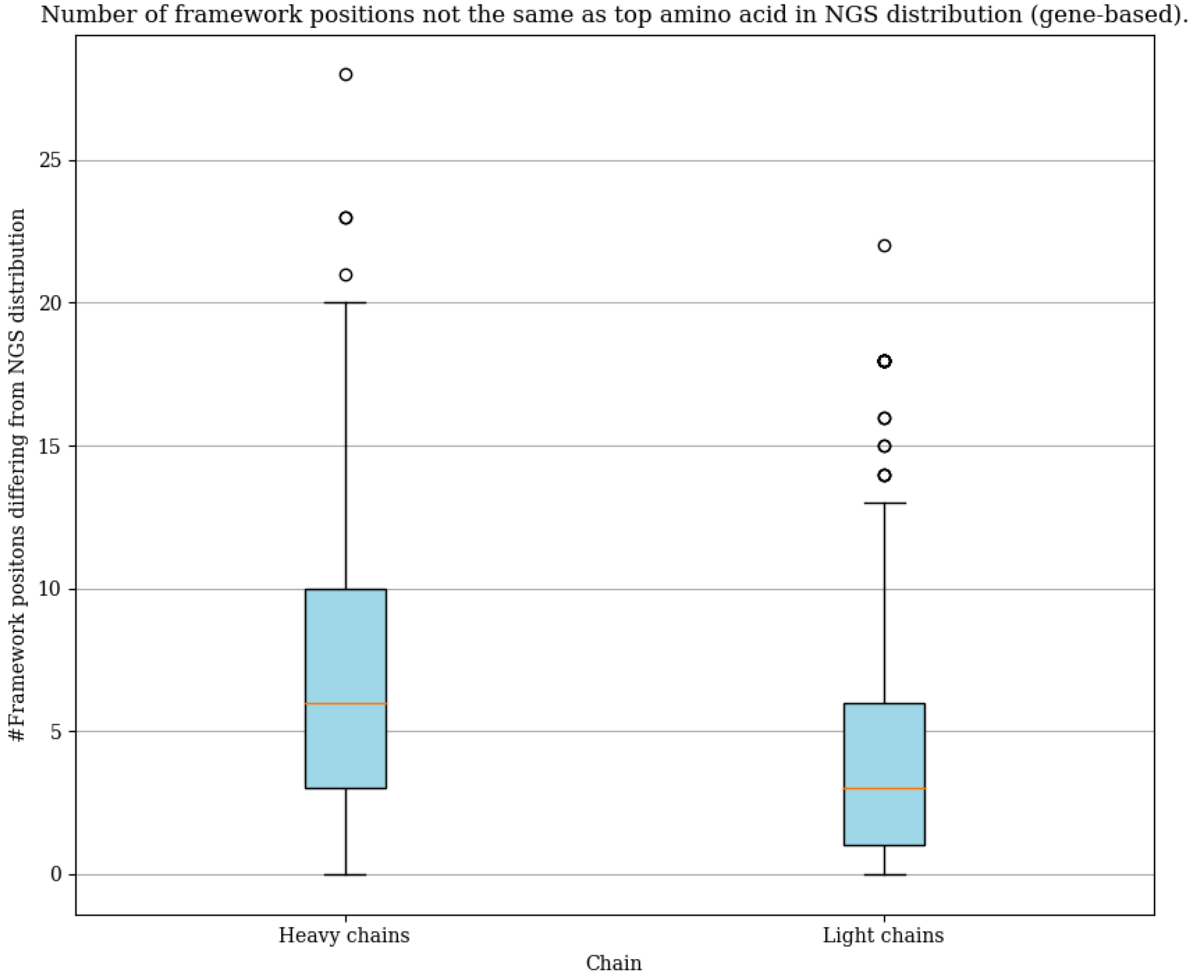

Supplementary Figure 3. Number of framework positions not agreeing with top amino acids in gene-based profiles.

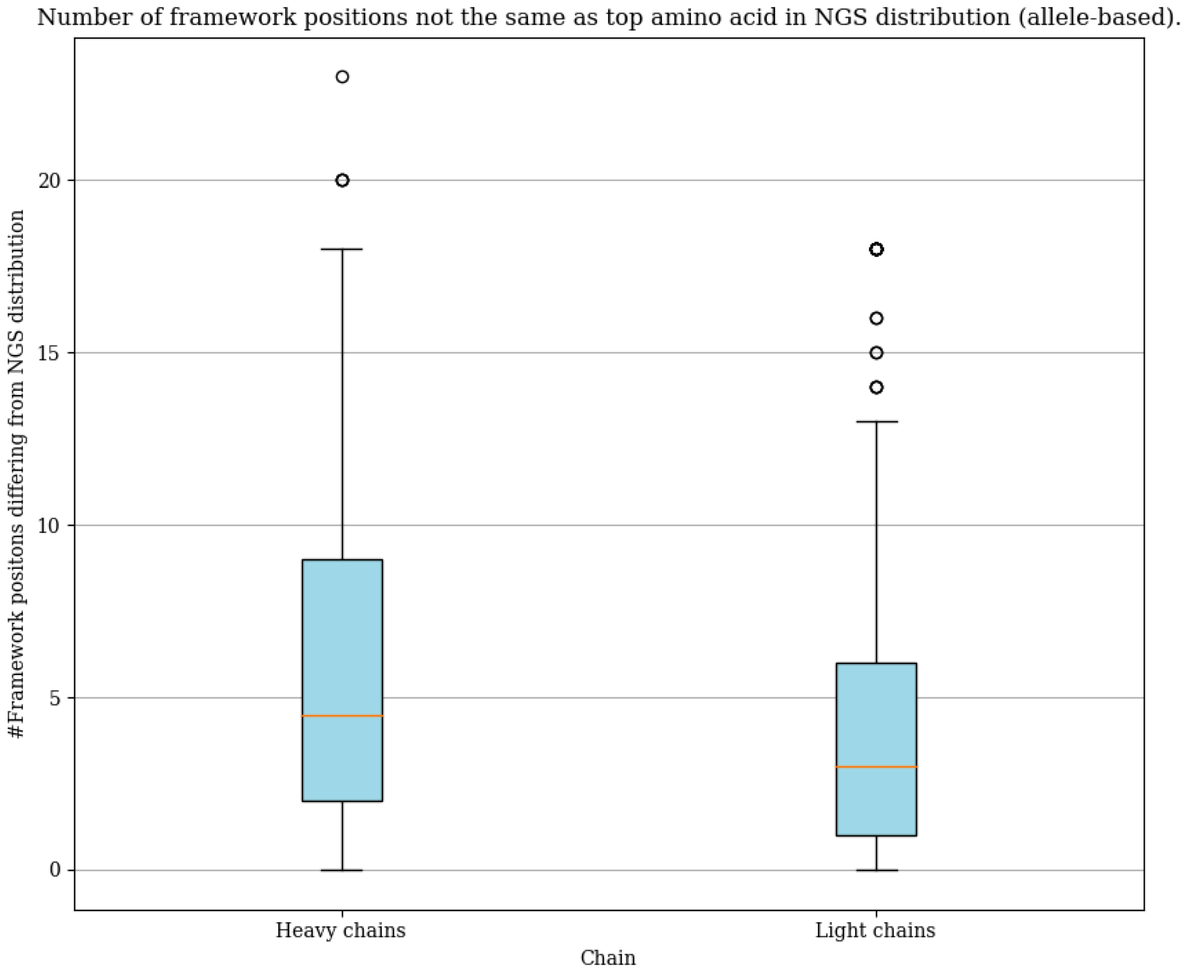

Supplementary Figure 4. Number of framework positions not agreeing with top amino acids in allele-based profiles.

Section 3. Sequence retrieval benchmark.

We benchmarked the speed of our search service. Retrieval time for AbDiver was calculated as time (in seconds) taken from issuing a query to the database, to returning the top 100 results. We benchmarked retrieval of the entire variable region (heavy and light separately) as well as CDR3 retrieval (CDR-H3 and CDR-L3 separately) for 742 therapeutic antibodies. The benchmark, expressed in seconds is given in Supplementary Figure 5. Mean retrieval time for full sequences was 1.56s for light chains and 4.40s for heavy chains. For CDR3 retrieval, the average time for CDR-L3 clones was 1.24s and for CDR-H3 clones it was 2.68s.

We benchmarked the ability of the V-region retrieval service to retrieve highly similar matches with sequence identity 90% or greater. We found 189/738 (25.60%) heavy chains that match greater than 90% sequence identity, with 4/738 (0.54%) perfect matches (Dusigitumab, Edrecolomab, Melredableukin and Zanolimumab). We find 288/707 (40.73%) light chains with sequence identity 90% or greater and 50/707 (7.07%) perfect matches. Therefore, despite restrictive length constraints that produces more relevant results, AbDiver identifies high quality sequence matches.

We benchmarked the ability of our clonotype service to retrieve sequences sharing the same V gene and with CDR3 sequence identity with the query of at least 80% (Greiff *et al.*, 2015). For 409/686 (59.62%) therapeutic heavy chains with unique CDRH3s we found matches greater than 80% sequence identity and for 172/686 (25.07%) matches greater than 90%. In 35/686 (5.10%) instances, a combination of V gene and CDRH3 can be matched to an identical CDRH3 and V gene of an NGS sequence. For 384/573 (67.01%) therapeutic light chains with unique CDRL3s we found matches greater than 80% sequence identity and for 279/573 (48.69%) matches greater than 90% with 244/573 (42.58%) perfect matches in V gene and CDRL3 sequence. Therefore, our service succeeds at finding high number of relevant matches for most of the therapeutics in our dataset.

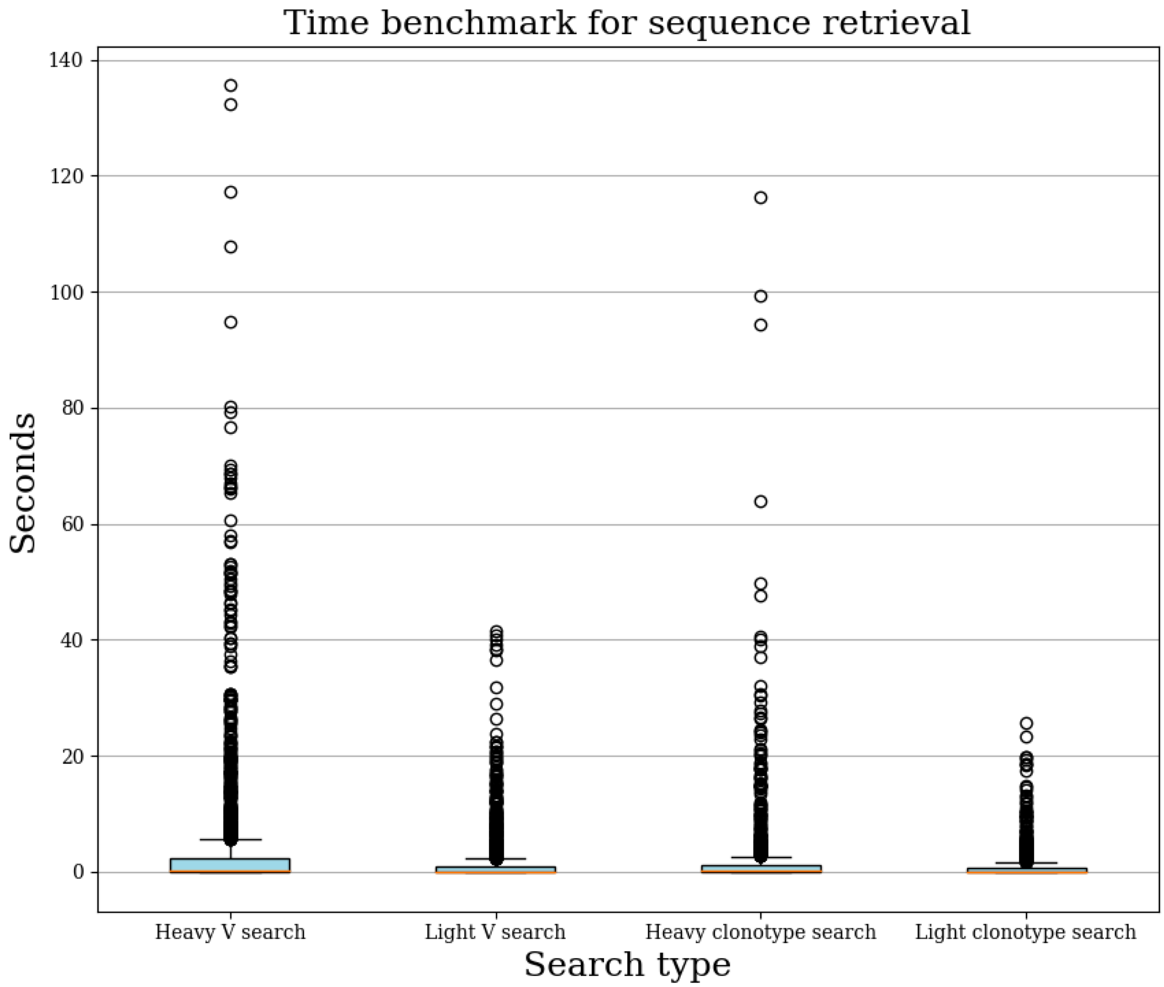

Supplementary Figure 5. Retrieval times statistics. For each chain and search type, the retrieval corresponds to wall clock time from issuing the query to when all the results were processed and returned.

References

Deszy´nski,P. *et al.* (2022) INDI—integrated nanobody database for immunoinformatics. *Nucleic Acids Res.*

Dunbar,J. and Deane,C.M. (2015) ANARCI: Antigen receptor numbering and receptor classification. *Bioinformatics*

Greiff,V. *et al.* (2015) Bioinformatic and Statistical Analysis of Adaptive Immune Repertoires. *Trends Immunol.*

Kovaltsuk,A. *et al.* (2018) Observed Antibody Space: A Resource for Data Mining Next-Generation Sequencing of Antibody Repertoires. *J. Immunol.*

Lefranc,M.P. (2011) IMGT unique numbering for the variable (V), constant (C), and groove (G) domains of IG, TR, MH, IgSF, and MhSF. *Cold Spring Harb Protoc.*

Smakaj,E. *et al.* (2020) Benchmarking immunoinformatic tools for the analysis of antibody repertoire sequences. *Bioinformatics*.

Steinegger,M. and Söding,J. (2018) Clustering huge protein sequence sets in linear time. *Nat. Commun*.
